# Patterns of Intergenerational Care Planning Among Chinese Aging Families in Hong Kong

**DOI:** 10.1093/geroni/igab046.2035

**Published:** 2021-12-17

**Authors:** Xue Bai, Chang Liu, Tongling Xu

**Affiliations:** The Hong Kong Polytechnic University, Hong Kong, Hong Kong

## Abstract

Care planning before the onset of intensive care needs can increase families’ ability to manage caregiving crises and cope with care transitions. However, future care planning has not been substantially examined in a family context. Drawing on the model of Preparation for Future Care Needs and a family systems perspective, this study investigated patterns of intergenerational care planning across multiple planning domains (awareness, avoidance, information gathering, decision making, and concrete planning) among Chinese intergenerational pairs. Quantitative data of 213 pairs of aging parents and adult children were collected in Hong Kong. Latent Profile Analysis was conducted to examine typological structure underlying care planning patterns. Three patterns were discovered: filial-maximal, dyadic-moderate, and filial-minimal. Profile 1 contained approximately 9.9% of pairs, which demonstrated a relatively higher level of avoidance on considering the need of care preparation and engaged less in concrete planning activities. Profile 2 contained 68.5% of intergenerational pairs that had a moderate preparation level. Profile 3 contained 21.6% of intergenerational pairs that were comparatively active in care planning. The findings also indicated that although older adults across three groups demonstrated a similar level of awareness to prepare for future care, their engagement in the concrete planning activities may be driven by their children’s awareness and preparation toward future care. The findings will enhance professionals’ and service providers’ awareness of diverse care planning patterns among Asian aging families, and inform targeted policies and programmes to alleviate unpreparedness for eldercare through intergenerational care planning which can be more effective than unilateral preparation.

